# NEWS

**DOI:** 10.7189/jogh.02.020203

**Published:** 2012-12

## DEMOGRAPHY

• In July 2012, the Bill & Melinda Gates Foundation and the UK Government Department for International Development (DFID) hosted an international summit on family planning. About 150 leaders from both low–income and donor countries, NGOs and the private sector committed to spend an additional US$ 4.3 billion on voluntary family planning. More than 20 developing countries pledged to address barriers to accessing family planning services. These actions could result in reduction of maternal deaths globally by 200 000, with a decrease in unintended pregnancies by about 110 million. The impact of those actions could also affect abortions, decreasing their number by about 50 million, and also reduce infant mortality by further 3 million by the year 2020. Accountability and monitoring of the commitments will be arranged to comply with the UN General Secretaries “Every Woman, Every Child” process, while the progress will be reviewed each year by an Independent Expert Review Group. (*BMGF*, 11 Jul 2012)

• In a recent five–part series in the *Los Angeles Times*, Ken Weiss explained the adverse effects of population growth, which are both well documented and wide–ranging. However, population reduction through dramatic fertility declines may also have unintended consequences for populations and their well-being, which are complex and require proper anticipating policies to address them. Despite major fertility declines in recent decades in many parts of the world, human population growth is set to continue. (*Center for Global Development*, 17 Aug 2012)

• According to the survey of 1000 adults in the United Kingdom, ‘middle age’ now begins much later than previously thought – at the age of 55. The average age at which the period of life was perceived to start was 54 years and 347 days old. British people do not see themselves as ‘elderly’ until they approach 70 years of age. Some earlier studies were reporting that the start of middle age may be as early as 36. There are now more adults older than 65 years in the UK than there are children and adolescents under 16 years of age. (*BBC News*, 17 Sept 2012)

• Marking Universal Children's Day in November this year, UNICEF released a study forecasting just a 4% increase in the global population of children by 2025. It added that this is expected as a net result of decreases in fertility rates in the North, and further increases in many countries in the South. (*UN News*, 20 Nov 2012)

• In a recent study of more than 25 000 French men, both the total sperm count and the percentage of normal sperm per unit of semen fluid have fallen by over 30% between 1989 and 2005. While the average number of spermatozoa remained above the level of infertility, the study demonstrated a significant decline in sperm concentration and viability across the whole country. Researchers argued that a decline in fertility across Europe may be partly due to this unexplained phenomenon, with several other studies suggested that up to 1 in 5 European men may be infertile due to low sperm count. However, researchers continue to debate the evidence for falling fertility and further research is needed to strengthen the observation and identify the causes of this ‘sperm decline’. (*BBC*, 5 Dec 2012)

## ECONOMY

• According to the United Nations Development Programme's (UNDP) in Africa, the continent's growth could rise to 7% by the year 2015. The growth will mainly be fuelled by investors that are building or improving its infrastructure. Although presently the poorest continent, Africa reported strong growth rates of about 5% in recent years, which was second only to Asia and which generated interest from investors. (*Reuters*, 7 Aug 2012)

• Mr Rajiv Shah of the US Agency for International Development (USAID) said that an Open Source Development (OSD) model – connecting problem–solvers with local challenges – could be effective in tackling poverty, promoting democracy and reducing child mortality. Mr Shah argued that within a generation preventable child deaths could end, education could improve and extreme poverty could be reduced by nearly two–thirds. (*SciDev.Net*, 14 Aug 2012)

• Legatum Institute's ‘Prosperity Index’ assesses global wealth and well-being for 142 countries around the world. The Institute has been reporting it for 6 years. The index takes into account eight categories: economy, education, entrepreneurship and opportunity, governance, health, personal freedom, safety and security and social capital. In this year's list, the United States has not been ranked in the top ten countries for the first time due to a weakening performance in five of the eight categories. (*The Guardian*, 30 Oct 2012)

• The Organisation for Economic Co–operation and Development (OECD) said in Paris earlier this year that China will overtake the United States in the next four years and become the largest economy in the world. OECD also predicts that China's economy will be larger than the combined economies of the Eurozone countries by the end of 2012 already. (*The Guardian*, 9 Nov 2012)

• Surprisingly to many observers, France has been given a second downgrade of its sovereign debt rating by Moody’s, with the agency removing its ‘triple A’ ranking in November. This followed a similar move in January by Standard & Poor’s agency. The change in rating is explained by concerns that the country’s high level of public debt, which has recently risen above 90% of its gross national product, puts the country in danger of becoming yet another victim of the debt crisis in the Eurozone. (*Financial Times*, 19 Nov 2012)

## ENERGY

• Engineers from Solar Electric Light Company (Selco) have provided solar panels to over 135 000 homes in Karnataka state in India at the price of US$ 125. The panels produce enough power for two light bulbs and a socket and many poor people both them through bank loans. Savings in energy costs due to the panels should allow repayment of those loans. More than 300 million people in India still live without electricity. (*The Guardian*, 3 Jul 2012)

• In November this year, BP has admitted guilt on 14 criminal charges and agreed to pay US$ 4.5 billion penalty related to the fatal explosion of its rig Deepwater Horizon and the catastrophic oil spill that subsequently polluted the Gulf of Mexico. Mr Eric Holder, the attorney general, said that “...this marks the single largest criminal fine – US$ 1.25 billion – and the single largest total criminal resolution – US$ 4 billion – in the history of the United States”. Three BP officials were also charged for manslaughter and negligence in supervising the pressure tests on the well, while a senior official was charged with obstruction of Congress and lying about how much oil was gushing from the well. This criminal settlement is expected to only deal with some of the claims against BP for the oil spill in April 2010, while the penalties for the environmental damage caused to the Gulf of Mexico could amount to as much as US$ 21 billion under the clean water act for restoration costs to waters, coastline and marine life. (*The Guardian*, 15 Nov 2012)

• United Kingdom may be leading the world in the proportion of energy derived from wind, but the public is deeply divided over their attitude to windfarms: proponents see UK as “the Saudi Arabia of wind”, and think of this as a comparative advantage which should be exploited to the full. Those who oppose them say that windfarms destroy the environment and cause more carbon dioxide damage than they relieve with the infrastructure that they demand – such as the extra roads and the turbines themselves, and that the cost of supplementing wind energy with gas–fired generators leads to greater carbon emissions than a gas turbine operating alone. They say windfarms kill birds and interfere with people's hearing and health, while those least convinced believe that wind energy is somewhat pointless because it can't be stored. (*The Guardian*, 30 Nov 2012)

• Mr Eyal Aronoff, the software entrepreneur, is a co–founder of the Fuel Freedom Foundation. This organisation's aim is cutting America's oil consumption, both foreign and domestic. Their philosophy is that if America cuts its use of oil in half over the next 10 years, prices on the global market would drop below US$ 50 per barrel to reflect this reduced demand, which would reduce the cost of gas to only US$ 2 per gallon. The foundation proposes that the initial reduction in oil dependence would come from widespread adoption of other liquid fuels for fuelling vehicles, such as petrol, ethanol, natural gas or methanol, which are all considerably cheaper. The foundation is against subsidizing cleaner fuels, thus “...turning conventional environmental thinking on its head”. (*The Guardian*, 10 Dec 2012)

• European Commission awarded about US$ 50 million to two renewable energy schemes planned in the west of Scotland. Tidal turbines should be installed between the Isle of Skye and the mainland for about half of that award, while Scottish Power Renewables plan to put another tidal array into the Sound of Islay. A total of 23 grants are being awarded throughout Europe and they are worth nearly US$ 1.5 billion. The projects range from Swedish biomass burning to solar power in Cyprus. The Commission did not fund any bids for carbon capture and storage because the bidders did not manage to secure backing by either industry or the governments. (*BBC News*, 18 Dec 2012)

## ENVIRONMENT

• The talks at the global UN Sustainable Development Summit in Rio were at risk of collapse, with opposing delegations still failing to reach agreement on the goals for our plant’s challenges. Tensions have been rising between the developing nations and negotiators from the US, EU, Switzerland and Korea, with provision for “additional financial resources” to sustain a green economy being a key stumbling block that was vetoed by the developed nations. Consequently, speculations have been rising that any policy derived from Brazil’s summer summit would be wearingly insubstantial to what many see as the mountainous task of sustainable global development. (*The Guardian*, 8 Jun 2012)

• Risk analysis firm Maplecroft has recently presented their “Natural Hazards Risk Atlas”. The publication shows that the emerging economies in Asia, such as India and the Philippines, are at the greatest financial risk from natural disasters. The firm assessed that the last year was the most costly on record for natural disasters, which caused damages estimated to approach US$ 380 billion. (*BBC* News, 15 Aug 2012)

• Using an analogy with financial markets, US–based marine researchers have created an index that assesses overall ocean vitality. The index, described in their study published by the Nature journal, comprises ten disparate measures. Those measures are then aggregated into a single score and they assess features such as food provision, carbon storage, tourism value and biodiversity, thus reflecting both the needs of humans and ecosystem sustainability. The index suggests that a global score is 60 out of 100, offering a seemingly gloomy picture. (*Nature*, 15 Aug 2012)

• In August 2012, the fate of billions of dollars promised by the governments of high–income countries to help the low– and middle–income countries to adapt to climate change was discussed in Geneva, at the first meeting of the UN's Green Climate Fund. It is unclear whether the GCF would have access to much financing in the first years of its existence, although it is envisaged as the world's single largest source of financing for climate change mitigation and adaptation by 2020. (*The Guardian*, 23 Aug 2012)

• A panel convened by the UN warned in September this year at a meeting in Bangkok that the system known as “the clean development mechanism” (CDM) was in dire need of rescue. CDM is world's only global system of carbon trading. It was designed to give poor countries access to new green technologies, but it has “essentially collapsed”, which prevents future flows of finance to the developing world. It is thought that the collapse of CDM would make it harder to raise funds that could help developing countries cut carbon emissions. (*The Guardian*, 10 Sept 2012)

## FOOD, WATER AND SANITATION

• Land deals see Africa heading towards “hydrological suicide” according to GRAIN, an organisation that backs small farmers. Pointing to both the Nile and Niger River basins as examples, GRAIN’s recent report: ‘Squeezing Africa dry: behind every land grab is a water grab’, highlights the dangers behind the continent’s land deals. Countries along the Nile, including Ethiopia and Egypt, have already leased out land that exceeds the water available in the basin. The report cites Pakistan, India, California, Kazakhstan and Uzbekistan, where water is currently used at a rate far beyond what can be replenished, as proof that such large–scale mega–irrigation schemes are far from sustainable. With one third of Africans already living in water scarce environments, GRAIN asserts the effects of these land deals would amount to an environmental disaster. (*The Guardian*, 12 Jun 2012)

• Four years ago, a harmful combination of bad harvests, questionable trade policies and inadequate governance created a global food crisis that put millions of lives at risk. This summer’s extreme drought in the Midwest region of the United States is threatening worldwide commodity prices again, which may test the commitments made after the last crisis at a G8 summit in L’Aquila, Italy in 2009. In August this year, FAO reported that global rice paddy production is expected to be lower than expected this year, mainly owing to below–normal monsoon rains in India. (*Financial Times*, 6 Aug 2012)

• Mr Justin Forsyth, the chief executive of the charity Save the Children, highlighted malnutrition as the “...Achilles heel of development”. Mr Forsyth praised UK's Prime Minister, Mr David Cameron, for linking the end of the London Olympic Games and the presence of many world leaders at the event with hosting a “hunger summit” in Downing Street. (*The Guardian*, 12 Aug 2012)

• UN Environment Programme (UNEP) reported that more than 400 million Africans now live in water–scarce countries, 300 million still lack reasonable access to safe drinking water and up to 230 million defecate in the open. This brings into question the lack of action from African governments to implement integrated water management policies and reach their sanitation targets. A survey of officials in 40 African countries by UNEP suggested that lack of funding is not the main reason behind this situation. Some suggested that water and sanitation would receive greater interest from local politicians if the contribution of safe water to development could be measured and communicated better. (*The Guardian*, 30 Aug 2012)

• In September, the EU's audit watchdog reported than half of the European Union's projects to provide safe drinking water in Sub–Saharan Africa had failed. The report, conveyed by the European Court of Auditors, had been examining 23 projects that were co–funded by the EU in six African countries between 2001 and 2010. In a statement that followed this report, the European Commission disputed some of the auditor's findings, although acknowledging that the projects could have been run better. (*Reuters*, 28 Sep 2012)

## PEACE AND HUMAN RIGHTS

• The 2012 Global Peace Index (GPI) found that the world has reversed its three–year worsening trend to become more peaceful for the first time since 2009. All regions apart from the Middle East and North Africa improved their levels of peacefulness. The GPI was developed by the Institute for Economics and Peace to provide a quantitative measure of peace and conflict across the world’s nations using 23 indicators, ranging from a nation’s military expenditure to their reputation for respecting human rights. Among the reasons for recent changes were austerity–driven defence cuts, which created gains in indicators related to militarisation. Iceland and Somalia were ranked the most and the least peaceful countries, respectively. (*The Guardian*, 12 Jun 2012)

• In June 2012, Canada was named the best place to live as a woman among 20 of the world’s largest economies. The poll was carried out by Trust Law, one of the core humanitarian and journalistic programmes of the Reuters Foundation. It asked 370 experts in gender issues – including aid workers, health professionals and journalists – to consider their decision based on a number of key issues facing women across the globe. The indicators included the percentage of women in education or roles of public responsibility, the rate of domestic violence and maternal health. Canada claimed the top spot partly because of its commitment to policies promoting gender equality, with women currently accounting for two thirds of university graduates and one third of its federally appointed judges. India ranked lowest, with one of the highest early marriage rates in the world (44.5%). (*Reuters*, 13 Jun 2012)

• It is estimated that about 30% of global aid may be lost to corruption. At the Economic and Social Council's high–level panel on accountability and transparency, held in July this year, UN Secretary–General Ban Ki–moon highlighted the importance of combating corruption. In his words, neither peace and development, nor human rights “...can flourish in an atmosphere of corruption”. (*Devex*, 10 Jul 2012)

• A drive to get more children into school in low– and middle–income countries is losing momentum as aid for education stagnates. The education for all (EFA) global monitoring report, “Putting education to work”, said that with continuation of current trends the millennium development goal (MDG) of universal primary education by 2015 will be missed by a very substantial margin. (*The Guardian*, 16 Oct 2012)

• An international training institute will be teaching human rights campaigners online tactics to increase the impact of their messages and broadcasting. The institute is being set up in the Italian city of Florence. The first students, starting in 2013, will be drawn from human rights activists around the world. The courses are expected to arm them with the latest tools for digital dissent. The recent events in Northern Africa showed that nowadays protests are as likely to be using social networking as public demonstrations, with “street protests becoming Tweet protests”. Similarly, repressive regimes are using Facebook for hunting down their opponents. The protesters need to balance their secrecy and safety with their need to achieve the maximum public impact. The training centre is being set up by the European wing of the Robert Kennedy Center for Justice and Human Rights. (*BBC* News, 5 Nov 2012)

## SCIENCE AND TECHNOLOGY

• During the 1920s it was noted that women who had undergone cauterization of the cervix following childbirth were unlikely to develop cervical cancer. A recent study published in journal PNAS found that a discrete population of cells at the squamocolumnar junction of the cervix are targeted by the human papilloma virus (HPV), which is thought to be responsible for nearly all cases of cervical cancer. Similar cell populations may also be found to be critical in the development of other HPV associated cancers, such as some anal, vaginal, penile and throat cancers. It may be possible to prevent cervical cancer by ablating the junction cells by a relatively simple cyro–probe procedure, which could be an option in countries where screening is too expensive to implement. (*AFP*, 11 Jun 2012)

• Presently, are about 2.5 billion people in low– and middle–income countries own a mobile phone, with coverage nearly 100% in large countries such as the Philippines, Mexico and South Africa and 85% in Uganda. People in poorer nations are now well connected and their movements, habits and ideas are more transparent. This change provides unforeseen opportunities to monitor poor societies, particularly those scattered over large regions, and acquire data relevant to improved public policies. (*Financial Times*, 10 Aug 2012)

• Counterfeit medications are a serious and potentially life–threatening problem in many low– and middle–income countries. Two teams of US–based scientists have developed quick tests can identify such drugs before they cause harm, using chemically treated paper the size of a business card. Rubbing a pill on the paper and dipping it in water indicates suspicious ingredients through colour changes. (*Voice of America*, 21 Aug 2012)

• A disturbing proportion of high–impact papers whose results could never be replicated prompted scientific publishers to endorse a new initiative: authors of research papers that report major breakthroughs would be encouraged to get their results replicated by independent laboratories. These validation studies would earn authors a certificate and a second publication on replication, protect them from disclosing their results to competitors and save other researchers from pursuing further work on incorrect results. “The Reproducibility Initiative” would work through Science Exchange, based in Palo Alto, California – a commercial online portal that matches scientists with experimental service providers. (*Nature*, 24 Aug 2012)

• About one–third of the world's population now has access to the internet. In its new report, the International Telecommunications Union said that more remains to be done to achieve internet coverage targets as set out in the Millennium Development Goals. An interesting feature of the report is a notion that a “...strong linguistic shift is now taking place online”. With current trends, the number of users accessing the internet in languages other than English, primarily in Chinese, will overtake English language usage by 2015. (*Al Jazeera*, 24 Sep 2012)

The editors gratefully acknowledge the contribution of the members of Edinburgh University Global Health Society to the News section: Rachel Banfield, Vijna Hiteshna Boodhoo, Frances Barclay, Kate Booth, Rachel Burge, Yu Cao, Michael Charteris, Kevin Choi, Alexander Fullbrook, Jenny Hall, Ewan D. Kennedy, Nethmee S. Mallawaarachchi, Paul Motta, Rachel Siow, Vicky Stanford, Thomas Tolley and Ryan Wereski.

